# Sarcopenia predicts poor long-term survival but not postoperative complications in gastric cancer surgery: an 18-year retrospective cohort study

**DOI:** 10.1186/s12957-025-04120-6

**Published:** 2025-12-02

**Authors:** Johan Back, Tommi Järvinen, Ville Sallinen, Pauli Puolakkainen, Arto Kokkola

**Affiliations:** 1 Department of Abdominal Surgery, Helsinki University Hospital and University of Helsinki, Finland, PO Box 340, Meilahti Tower Hospital, Building 1, Haartmaninkatu 4, Helsinki, 00029 HUS Finland; 2 Department of General Thoracic and Oesophageal Surgery, Helsinki University Hospital, University of Helsinki, Helsinki, Finland; 3 Department of Transplantation and Liver Surgery, Helsinki University Hospital, University of Helsinki, Helsinki, Finland

**Keywords:** Sarcopenia, Complications, Gastric cancer

## Abstract

**Introduction:**

Sarcopenia, defined as the loss of skeletal muscle mass and function, is common in gastric cancer (GC) patients and has been associated with adverse outcomes. This study aimed to evaluate the impact of sarcopenia on postoperative complications, mortality, and long-term survival following gastrectomy for GC.

**Methods:**

We conducted a retrospective cohort study of 337 patients who underwent gastrectomy with curative intent at a single tertiary centre between 2000 and 2017. Sarcopenia was defined using CT-based skeletal muscle index at the L3 level, threshold values of ≤ 39 cm²/m² for women and ≤ 55 cm²/m² for men. The primary outcome was overall survival (OS). Postoperative complication burden assessed using the Comprehensive Complication Index (CCI), along with 30- and 90-day mortality and, disease-specific survival (DSS), and disease-free survival (DFS) formed secondary outcomes.

**Results:**

Sarcopenia was present in 174 (51.6%) and 163 (48.4%) patients were defined as non-sarcopenic. Complication burden, 30- and 90-day mortality, and DSS/DFS did not differ by sarcopenia status. In the overall cohort, sarcopenia was not independently associated with OS. However, a significant sarcopenia × stage interaction was observed: sarcopenia predicted poorer OS in stage 0–II patients (HR 1.82, 95% CI 1.05–3.17, *P* = 0.034) but not in stage III–IV disease.

**Conclusions:**

Sarcopenia was not associated with increased postoperative morbidity or short-term mortality, but in stage 0–II GC it was an adverse prognostic factor, conferring an 82% higher risk of death. Routine CT-based assessment may help identify high-risk early-stage patients who could benefit from timely nutritional and physical interventions.

**Supplementary Information:**

The online version contains supplementary material available at 10.1186/s12957-025-04120-6.

## Introduction

 Gastric cancer (GC) remains a major global health concern, with approximately 1 million new cases diagnosed annually, making it the fourth leading cause of cancer-related deaths worldwide [1]. Despite advancements in treatment, long-term survival remains poor, particularly in advanced stages. Surgical resection with adequate lymph node dissection continues to be the cornerstone of treatment for resectable GC [2, 3].

Sarcopenia, defined as the loss of skeletal muscle mass and function, is common in patients with GC due to poor nutritional intake and cancer-related metabolic alterations [4, 5]. Tumour-induced anorexia, dysphagia, malabsorption after gastrectomy, and systemic inflammation contribute to muscle wasting and reduced physical performance [5].

Patients with sarcopenia are more susceptible to postoperative complications, prolonged recovery, and extended hospital stays [5–7]. Moreover, sarcopenia has been associated with lower chemotherapy tolerance and poorer long-term survival, suggesting it may be an important prognostic factor in GC [5, 8, 9].

Although sarcopenia is generally associated with poor outcomes in gastric cancer, its relative impact may vary across tumour stages. In advanced disease, survival is largely determined by tumour burden and biology, which may mask the influence of host-related factors. In contrast, among patients with lower-stage disease—where long-term survival is otherwise achievable—sarcopenia may have a clearer prognostic effect. This study therefore examines the prognostic significance of sarcopenia and its association with surgical and oncological outcomes in patients undergoing gastrectomy for GC.

## Materials and methods

### Study design and patient selection

This retrospective study was conducted at Helsinki University Hospital (HUH), a teaching hospital that provides both secondary and tertiary care for GC surgery, serving a population of approximately 2.2 million. Patient identification was carried out using the electronic operating room database, based on the International Classification of Diseases (ICD-10) code C16, which denotes malignant neoplasm of the stomach, along with relevant procedure codes for gastrectomy (JCD10, JDA96, JDC00, JDC10, JDC20, JDC30, JDC96, JDD00, and JDD96). The data collection was limited to procedures performed between January 1, 2000, and December 31, 2017.

### Study endpoints

The primary endpoint was overall survival (OS). Secondary endpoints include disease-specific survival (DSS), and disease-free survival (DFS), along with comprehensive complication index (CCI), 30-day and 90-day mortality.

### Data collection

We manually extracted data from patient records, including demographic details, American Society of Anesthesiologists (ASA) classification, medication use, and comorbidities assessed using the Charlson Comorbidity Index [10]. Additionally, laboratory results, surgical characteristics, length of hospital stay, intensive care unit (ICU) admissions, reoperations, and readmissions within 30 days after discharge were collected. Postoperative complications were classified according to the Clavien–Dindo grading system [11], and the overall complication burden was evaluated using the Comprehensive Complication Index (CCI) [12]. The date of diagnosis, typically determined during gastroscopy, was also recorded. To ensure transparency, missing laboratory values were documented as missing, and no imputation was performed; analyses were conducted using complete-case data. Missing CT scans, height measurements, and laboratory results were primarily due to unavailable imaging or laboratory records from referring hospitals, which used separate electronic data systems.

### Pathological data

The pathological analysis classified tumour histology according to the Lauren classification [13] and determined staging based on the AJCC TNM 7th edition criteria [14]. Postoperative mortality was defined as death occurring within 30 days after gastrectomy. Follow-up data were manually retrieved from hospital patient records and the Population Information System. This system, maintained by the Finnish Digital and Population Data Services Agency maintains up-to-date records on whether a person is alive or deceased, ensuring accurate identification [15]. The most recent recorded patient contact with a healthcare facility, potential disease recurrence, long-term mortality, and cause of death were extracted from hospital records. For patients whose follow-up was conducted outside HUH, additional records were obtained from the referring hospital to ensure comprehensive follow-up data.

### Patient selection and recurrence follow-up

Curative intent was defined as the goal of achieving an R0 resection, as determined intraoperatively by the surgeon. An R0 resection was classified as the complete removal of all malignant tissue with histologically negative margins, typically verified through frozen section analysis during surgery. Patients with limited, resectable metastatic disease, such as a single metastatic lesion (e.g., ovarian metastasis), were included if an R0 resection was accomplished. However, those with unresectable or disseminated metastatic disease were excluded from the study.

Disease recurrence was diagnosed based on radiological findings (primarily CT scans), surgical exploration, or endoscopic evaluation. At our institution, follow-up imaging is performed selectively, based on clinical suspicion of recurrence, such as new symptoms or elevated tumour markers (e.g., CEA, CA19-9), rather than as part of routine surveillance.

### Definition of sarcopenia and patient grouping

All skeletal muscle measurements were obtained from preoperative staging CT scans performed before gastrectomy. A single axial CT image at the L3 vertebral level, ensuring visibility of both transverse processes, was selected for analysis. Images were processed using OsiriX Version 3.3 (32-bit Pixmeo, OsiriX, Sarl, Switzerland), where abdominal muscles were identified using a semi-automated region of interest (ROI) selection. Skeletal muscle tissue was defined within a Hounsfield Unit (HU) range of − 29 to + 150. Manual corrections were performed when necessary, using the propulsion and brush tools in OsiriX.

The cross-sectional total muscle area (TMA) at L3 was measured, and the skeletal muscle index (SMI) was calculated as TMA divided by height squared (cm²/m²). Sarcopenia was classified based on internationally validated consensus criteria, with threshold values of ≤ 39 cm²/m² for women and ≤ 55 cm²/m² for men [16]. As functional assessments such as handgrip strength were not routinely recorded during the study period, sarcopenia in this study reflects CT-defined low skeletal muscle index rather than the full EWGSOP criteria that also require low muscle strength.

### Perioperative chemotherapy

Perioperative EOX (epirubicin, oxaliplatin and capecitabine) chemotherapy was introduced in 2006 and was initially administered selectively, primarily to younger and fitter patients with locally advanced gastric cancer (e.g., cT3–T4 and/or cN+) based on preoperative staging [17, 18]. Over time, its use expanded, and by 2010, EOX had become the standard treatment for locally advanced gastric cancer, leading to routine adoption in clinical practice in our institution. However, in recent years, EOX has been progressively replaced by FLOT (fluorouracil, leucovorin, oxaliplatin and docetaxel) as the preferred perioperative chemotherapy regimen [19].

### Statistical analysis

All statistical analyses were conducted using SPSS^®^ version 29 (IBM, Armonk, NY, USA). Continuous variables were compared using the Student’s t-test for normally distributed data and the Mann–Whitney U-test for non-normally distributed data. Normally distributed continuous variables are presented as the mean (± SD), while non-normally distributed variables are reported as the median [interquartile range (IQR)]. Distributional characteristics were evaluated using summary statistics, and a non-parametric approach was preferred for variables that showed apparent skewness. Categorical variables were analysed using the chi-square test or Fisher’s exact test when expected frequencies were below five.

Survival outcomes were assessed with the Kaplan–Meier method, and differences between groups were determined using the log-rank test. Cox proportional hazards regression was applied to estimate hazard ratios (HRs) with 95% confidence intervals (CIs). Univariable models were first constructed, followed by multivariable models adjusted for clinically relevant covariates, including age, sex, body mass index (BMI), ASA class, Charlson comorbidity index, adjuvant treatment, type of gastrectomy, additional resections, histological subtype, pathological stage, and R-class. Because the prognostic effect of sarcopenia was hypothesised to differ by tumour stage, we tested an interaction term between sarcopenia and stage (0–II vs. III–IV) within the fully adjusted multivariable model. When a significant interaction was identified, stage-specific Cox analyses were performed, adjusted for sex and BMI. The proportional hazards assumption was assessed by visual inspection of log–log survival plots, which demonstrated roughly parallel curves for all variables, indicating that the assumption was reasonably satisfied.

Overall survival (OS) was defined as the time from diagnosis to death from any cause, with patients still alive at the last follow-up censored. Disease-specific survival (DSS) was measured from diagnosis to death specifically due to gastric cancer, censoring patients alive or deceased from other causes. Disease-free survival (DFS) was calculated from diagnosis to either recurrence or death from gastric cancer, with patients censored at the last follow-up if alive without recurrence or deceased from unrelated causes. Median survival times were reported when available. For outcomes where the median was not reached, 5-year Kaplan–Meier survival estimates were presented. All statistical tests were two-tailed, with *P*-values below 0.05 considered statistically significant.

## Results

### Patient cohort

Between January 1, 2000, and December 31, 2017, a total of 420 patients underwent gastrectomy with curative intent for GC. Of these, 66 patients were excluded due to missing data (e.g., preoperative CT scans or height measurements), and an additional 17 patients were excluded having undergone emergency surgery, which resulted in a final study cohort of 337 patients. Based on the skeletal muscle index (SMI), 174 patients were classified as sarcopenic, while 163 were categorized as non-sarcopenic. The baseline characteristics of the study population are presented in Table 1 and operative details in Table 2, a detailed flowchart of the inclusion and exclusion process is provided in Supplementary Material 1.

**Table 1:** Baseline characteristics

	All patients *N* = 337	Sarcopenic *N* = 174	Non-Sarcopenic *N* = 163	*P*
Median age, in years (IQR)	68.5 (60.3–76.6)	69.6 (62.0–76.8.0.8)	66.7 (57.3–76.2)	0.074
Sex, male (%)	178	117 (67.2)	61 (37.4)	< 0.001
Mean body mass index, in kg/m^2^ (± SD)	25.7 (5.0)	23.8 (3.7)	27.7 (5.5)	< 0.001
ASA physical status, n (%)				0.521
1	26	12 (6.9)	14 (8.6)	
2	99	46 (26.4)	53 (32.5)	
3	175	95 (54.6)	80 (49.1)	
4	37	21 (12.1)	16 (9.8)	
Comorbidities, n (%)				
Myocardial infarction	21	13 (7.5)	8 (4.9)	0.331
Congestive heart failure	21	10 (5.7)	11 (6.7)	0.704
Peripheral vascular disease	15	11 (6.3)	4 (2.5)	0.085
Cerebrovascular disease	25	16 (9.2)	9 (5.5)	0.198
Hemiplegia	10	6 (3.4)	4 (2.5)	0.752
COPD or asthma	29	18 (10.3)	11 (6.7)	0.239
Connective tissue disease	11	4 (2.3)	7 (4.3)	0.303
Liver disease				
Mild	4	4 (2.3)	0	0.123
Moderate	1	1 (0.6)	0	1.000
Diabetes mellitus				
without complications	49	20 (11.6)	29 (17.8)	0.106
with complications	6	4 (2.3)	2 (1.2)	0.686
Kidney disease (moderate/severe)	13	10 (5.7)	3 (1.8)	0.063
Cancer (metastatic)	12	5 (2.9)	7 (4.3)	0.482
Peptic ulcer	28	19 (10.9)	9 (5.5)	0.073
Lymphoma	1	1 (0.6)	0	1.000
Dementia	8	5 (2.9)	3 (1.8)	0.724
Charlson comorbidity index, n (%)				
mild (0–2)	177	86 (49.7)	91 (55.8)	0.262
moderate (3–4)	117	63 (36.2)	54 (33.1)	0.553
Severe (≥ 5)	42	24 (13.8)	18 (11.0)	0.445
Mean Charlson comorbidity index (± SD)	3.0 (1.4)	3.1 (1.5)	2.8 (1.3)	0.157
Medication, n (%)				
Anticoagulant	27	15 (8.6)	12 (0.6)	0.671
Corticosteroid	15	10 (5.7)	5 (3.1)	0.233
Immunosuppressive	2	1 (0.6)	1 (0.6)	1.000
Laboratory, median (IQR)				
CA19-9	8.0 (4.0–17.0)	8.0 (4.0–16.3.0.3)	8.0 (3.0–17.0)	0.545
Missing no	70	36	34	
CEA	2.1 (1.3–3.4)	2.1 (1.3–3.3)	2.2 (1.4–3.6)	0.268
Missing no	42	22	20	
Hb	125.0 (114.0–137.0.0.0)	124.0 (112.5–136.0)	127.5 (116.0–139.0.0.0)	0.177
Missing no	2	1	1	
Alb	37.6 (34.3–39.7)	37.5 (34.0–39.4.0.4)	37.8 (34.9–39.9)	0.141
Missing no	8	5	3	
Creatinine	71.0 (60.0–82.0)	71.0 (61.0–85.0)	70.5 (59.8–79.0)	0.250
Missing no	2	1	1	
Neoadjuvant treatment (%)	101	57 (32.8)	44 (27.0)	0.248

*Abbreviations*: *IQR* interquartile range, *SD* standard deviation, *ASA* American Society of Anesthesiologists class, *COPD* chronic obstructive pulmonary disease, *CEA* carcinoembryonic antigen, *CA 19 − 9* cancer antigen 19 − 9 Hb, haemoglobin, *Alb* albumin

Continuous variables are presented as *mean ± SD* or *median (IQR)*, and categorical variables as *n (%)*

**Table 2:** Operative details

	All patients *N* = 337	Sarcopenic *N* = 174	Non-Sarcopenic *N* = 163	*P*
Type of resection, n (%)				
Total gastrectomy	171	95 (54.6)	76 (46.6)	0.144
Distal gastrectomy	166	79 (45.4)	87 (53.4)	
Approach, n (%)				
Open	316	164 (94.3)	152 (93.3)	0.704
Laparoscopic*	21	10 (5.7)	11 (6.7)	
Lymph node dissection, n (%)				0.989
D0	2	1 (0.6)	1 (0.6)	
D1	44	23 (13.2)	21 (12.9)	
D1+	24	13 (7.5)	11 (6.7)	
D2	267	137 (78.5)	130 (79.8)	
Time median, in minutes (IQR)	208 (173–243)	204 (172–236)	214 (175–248)	0.216
Blood loss (ml), median (IQR)	500 (300–900)	500 (300–900)	500 (300–900)	0.267
Resection of adjacent or another organ**				
Splenectomy	107	54 (31.0)	53 (32.5)	0.815
Liver	2	2 (1.1)	0	0.266
Pancreas	15	6 (3.4)	9 (5.5)	0.433
Other	18	9 (7.3)	9 (7.4)	1.000
Reconstruction, n (%)				0.081
Roux-en-Y	187	101 (58.0)	86 (52.8)	
Billroth I	7	6 (3.4)	1 (0.6)	
Billroth II	143	67 (38.5)	76 (46.6)	

*Abbreviations*: *IQR* interquartile range

Continuous variables are presented as *median (IQR)*, and categorical variables as *n (%)*

*Conversions are in the open category

**This included the following resections: 6 transverse colon resections, 1 rectum resection, 3 bowel resections, 3 adrenal gland resections, 1 kidney resection, 1 breast resection, 1 esophageal resection and 3 ovary resections

### Demographics and clinical characteristics

Sarcopenic patients were slightly older than non-sarcopenic patients (median 69.6 vs. 66.7 years, *P* = 0.074). Sarcopenia was significantly more common in males (67.2% vs. 37.4%, *P* < 0.001) and associated with a lower mean BMI (23.8 vs. 27.7 kg/m², *P* < 0.001).

### ASA classification and comorbidities

ASA classification was similar between groups (*P* = 0.521), with most patients classified as ASA class 3. Comorbidities were also comparable. Peripheral vascular disease (6.3% vs. 2.5%, *P* = 0.085) and moderate/severe kidney disease (5.7% vs. 1.8%, *P* = 0.063) were more frequent in sarcopenic patients, though not statistically significant. Charlson Comorbidity Index scores were slightly higher in sarcopenic patients (mean 3.1 vs. 2.8, *P* = 0.157), but overall distribution did not differ (*P* = 0.262), with mild comorbidity being most common.

### Medication and laboratory values

The use of anticoagulants, corticosteroids, and immunosuppressive medications was similar between the two groups, with no significant differences observed. Laboratory values, including CA19-9, carcinoembryonic antigen (CEA), haemoglobin (Hb), albumin (Alb), and creatinine levels, were also comparable between sarcopenic and non-sarcopenic patients, with no statistically significant differences.

### Neoadjuvant treatment

A higher proportion of sarcopenic patients received neoadjuvant treatment compared to non-sarcopenic patients (32.8% vs. 27.0%), although this difference was not statistically significant (*P* = 0.248).

### Operative details

Total gastrectomy was performed in 54.6% of sarcopenic patients compared to 46.6% in non-sarcopenic patients (*P* = 0.144), while surgical approach did not differ significantly, with most procedures conducted via open surgery. D2 lymphadenectomy was the most common extent of dissection in both groups (78.5% vs. 79.8%, *P* = 0.989).

Median operative time was 204 min in sarcopenic patients vs., 214 min in non-sarcopenic patients (*P* = 0.216), while blood loss was comparable (median 500 mL in both groups, *P* = 0.267). Splenectomy was performed in roughly one-third of patients in both groups (31.0% vs. 32.5%, *P* = 0.815). Other adjacent organ resections included pancreas (3.4% vs. 5.5%), liver (1.1% vs. 0%), and various others such as bowel, adrenal gland, kidney, breast, oesophagus, and ovary, with no significant differences observed.

### Operative results and complications

Postoperative outcomes were comparable between groups (Table 3). Complication severity did not differ (Clavien–Dindo, *P* = 0.637), and median CCI was identical (median 22.6 in both groups, *P* = 0.782). Reoperation, ICU admission, and readmission rates were similar. Median hospital stay was 9 days in the sarcopenic vs. 8 days in the non-sarcopenic group (*P* = 0.631). Thirty-day mortality was 1.1% in sarcopenic and 0.6% in non-sarcopenic patients (*P* = 1.000); 90-day mortality was 2.3% vs. 0.6% (*P* = 0.373), respectively.

**Table 3:** Operative results

	All patients *N* = 337	Sarcopenic *N* = 174	Non-sarcopenic *N* = 163	*P*
Clavien–Dindo grade, n (%) *				0.637
0	45	22 (12.6)	23 (14.1)	
1	83	42 (24.1)	41 (25.2)	
2	133	69 (39.7)	64 (39.3)	
3a	46	26 (14.9)	20 (12.3)	
3b	10	6 (3.4)	4 (2.5)	
4a	10	6 (3.4)	4 (2.5)	
4b	7	1 (0.6)	6 (3.7)	
5	3	2 (1.1)	1 (0.6)	
CCI, median (IQR)	22.6 (8.7–33.2)	22.6 (8.7–33.2)	22.6 (8.7–33.6)	0.782
Reoperation, n (%)	17	10 (5.7)	7 (4.3)	0.623
ICU admission, n (%)	39	20 (11.5)	19 (11.7)	1.000
Length of hospital stay (days), median (IQR)	9 (7–12)	9 (7–12)	8 (7–11)	0.631
Readmission, n (%)	36	20 (11.5)	16 (9.8)	0.725
30-day mortality, n (%)	3	2 (1.1)	1 (0.6)	1.000
90-day mortality, n (%)	5	4 (2.3)	1 (0.6)	0.373

*Abbreviations*: *IQR* interquartile range, *CCI* comprehensive complication index, *ICU* intensive care unit

Continuous variables are presented as *median (IQR)*, and categorical variables as *n (%)*

* Highest complication grade

### Pathological results

Pathological characteristics were largely comparable between sarcopenic and non-sarcopenic patients (Table 4). Histological subtype distribution did not show significant differences (*P* = 0.174), with diffuse-type carcinoma being the most common in both groups (51.7% vs. 62.0%). Tumour location differed between groups (*P* = 0.005), with upper gastric tumours more frequently observed in sarcopenic patients (9.8% vs. 3.7%). Pathological T category distribution was similar (*P* = 0.083), and lymph node status showed no significant difference (55.2% vs. 54.0% positive nodes, *P* = 0.913). The median number of lymph nodes harvested was 20 in both groups (*P* = 0.495), and the median number of metastatic lymph nodes was 1 in both groups (*P* = 0.956).

**Table 4:** Pathological details

	All patients *N* = 337	Sarcopenic *N* = 174	Non-sarcopenic *N* = 163	*P*
Histology, n (%)				0.174
Intestinal	115	66 (37.9)	49 (30.1)	
Diffuse	191	90 (51.7)	101 (62.0)	
Mixed/other*	31	18 (10.3)	13 (8.0)	
Tumour location, n (%)				0.005
Lower (antrum, angulus)	125	62 (35.6)	63 (38.7)	
Middle (body)	178	85 (48.9)	93 (57.1)	
Upper (fundus, cardia)	23	17 (9.8)	6 (3.7)	
Other**	11	10 (5.7)	1 (0.6)	
pT category, n (%)				0.083
pT0***	14	10 (5.7)	4 (2.5)	
pT1	72	30 (17.2)	42 (25.8)	
pT2	65	29 (16.7)	36 (22.1)	
pT3	102	59 (33.9)	43 (26.4)	
pT4	84	46 (26.4)	38 (23.3)	
Lymph node status, n (%)				0.913
Negative	153	78 (44.8)	75 (46.0)	
Positive	184	96 (55.2)	88 (54.0)	
cM category, n (%)	11	5 (2.9)	6 (3.7)	0.765
Lymph node harvested, median (IQR)	20 (14–30)	20 (14–31)	20 (13–29)	0.495
Stage, n (%)				0.784
0	12	8 (4.6)	4 (2.5)	
IA	55	24 (13.8)	31 (19.0)	
IB	40	20 (11.5)	20 (12.3)	
IIA	55	27 (15.5)	28 (17.2)	
IIB	42	25 (14.4)	17 (10.4)	
IIIA	39	23 (13.2)	16 (9.8)	
IIIB	59	29 (16.7)	30 (18.4)	
IIIC	23	12 (6.9)	11 (6.7)	
IV	11	5 (2.9)	6 (3.7)	
Metastatic lymph nodes, median (IQR)	1 (0–5)	1 (0–4)	1 (0–5)	0.956
Tumour size (mm), median (IQR)	40.0 (25.0–70.0)	45.0 (25.0–70.0)	35.0 (20.0–75.0)	0.169
R class, n (%)				0.268
R0	320	163 (93.7)	157 (96.3)	
R1	16	11 (6.3)	6 (3.7)	
Adjuvant treatment (%)	128	65 (37.4)	63 (38.7)	0.612
Missing no	1	0	1	

*Abbreviations*: *IQR* interquartile range

Continuous variables are presented as *median (IQR)*, and categorical variables as *n (%)*

*Histology is classed as ‘other’ when the histology could not be determined from the gastroscopy biopsies and the final specimen showed no tumour due to a total response of neoadjuvant treatment

**Seven cases had cancer in the remnant ventricle due to a previous gastric resection, four cases had cancer affecting the whole ventricle

***10 cases of total response due to neoadjuvant treatment. One patient had GC in the gastroscopy biopsies, but this was not confirmed in the specimen by pathology

Tumour stage distribution did not significantly differ (*P* = 0.784), and tumour size was comparable, with a median of 45 mm in sarcopenic and 35 mm in non-sarcopenic patients (*P* = 0.169). Resection status (R0 93.7% vs. 96.3%, *P* = 0.163) was also similar.

### The administration of pre- and postoperative chemotherapy

The administration of neo- and adjuvant treatment was comparable between sarcopenic and non-sarcopenic patients. In the sarcopenic group, 57 (32.8%) patients received preoperative EOX. Of these, 50 (87.7%) completed three or more cycles, while 3 patients received two cycles, and 4 patients received only one cycle. Postoperatively, 65 (37.4%) patients received adjuvant treatment in the sarcopenic group.

Among non-sarcopenic patients, 44 (27.0%) received preoperative EOX, while 37 (84.1%) completed all three or more cycles, while 3 patients received two cycles, and 4 patients received only one cycle. The remaining patients received one (4 patients), two (3 patients) cycles of EOX preoperatively. Postoperatively, 63 (38.7%) received adjuvant treatment in the non-sarcopenic group.

### Long-term survival

**Fig. 1:**
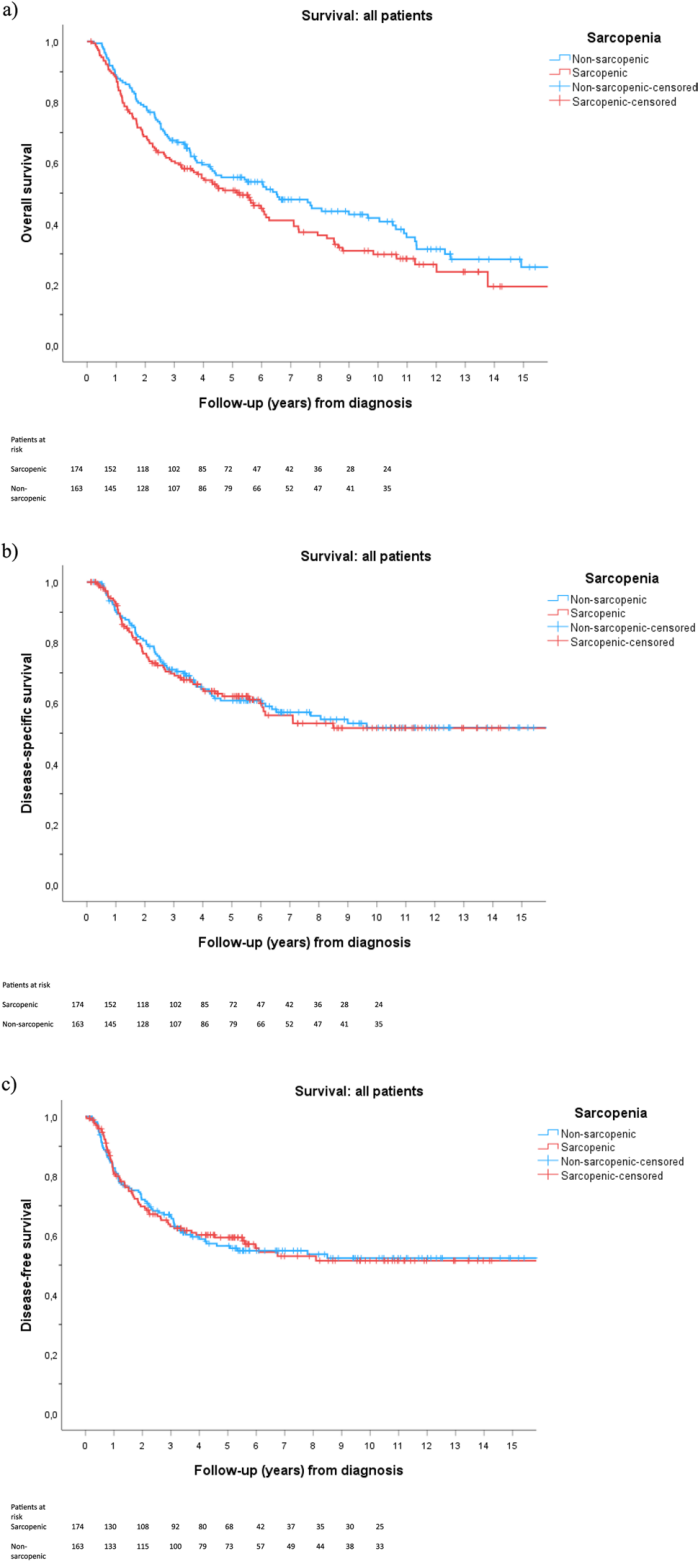
Kaplan-Meier survival of all patients (**a**) Overall survival (OS, *P* = 0.101); (**b**) disease-specific survival (DSS, *P* = 0.867) and (**c**) disease-free survival (DFS, *P* = 0.959). Number-at-risk tables are displayed below the X-axis

Survival between sarcopenic and non-sarcopenic patients is presented in Fig. 1. The median follow-up from diagnosis was 3.9 years (IQR 1.7–7.8) for all patients, 3.6 years (IQR 1.3–6.1) for sarcopenic patients, and 4.3 years (IQR 2.0–8.9) for the non-sarcopenic patients (*P* = 0.023).

The median estimated overall survival (OS) from diagnosis was 5.7 years (95% confidence interval [CI], 6.9–8.4). Among sarcopenic patients, the median OS was 5.2 years (95% CI, 3.8–6.7), compared to 6.5 years (95% CI, 4.1–9.0) in non-sarcopenic patients. Five-year OS was 50.9% in sarcopenic patients and 55.2% in non-sarcopenic patients (*P* = 0.101).

Median survival was not reached for either group in disease-specific survival (DSS) or disease-free survival (DFS). Five-year DSS was 62.2% in sarcopenic patients and 60.8% in non-sarcopenic patients (*P* = 0.867), while five-year DFS was 59.3% and 56.4%, respectively (*P* = 0.959).

In univariable Cox regression, sarcopenia was associated with a non-significant increase in the risk of death (HR 1.25, 95% CI 0.95–1.64, *P* = 0.116, Supplementary Material 2a).

In multivariable Cox regression, sarcopenia was not independently associated with survival (HR 1.03, 95% CI 0.75–1.42, *P* = 0.860, see Supplementary Material 2b). As expected, advanced stage (III or above) was the strongest predictor of poor prognosis (HR 3.21, 95% CI 2.35–4.39, *P* < 0.001). Other independent predictors included total gastrectomy, higher ASA class, and older age, whereas receipt of postoperative chemotherapy was associated with improved survival.

### Stage-specific survival

Because the prognostic effect of sarcopenia was hypothesized to differ by tumour stage, we tested an interaction between sarcopenia and stage (0–II vs. III–IV). This was statistically significant (*P* = 0.034), indicating that the effect of sarcopenia varied by stage. In stage-specific analyses, sarcopenia was associated with an 82% increased risk of death among patients with stage 0–II disease (HR 1.82, 95% CI 1.05–3.17, *P* = 0.034; Supplementary Material 2c), whereas no association was observed in stage III–IV patients. Corresponding stage 0–II subgroup characteristics are presented in Supplementary Material 3.

**Fig. 2:**
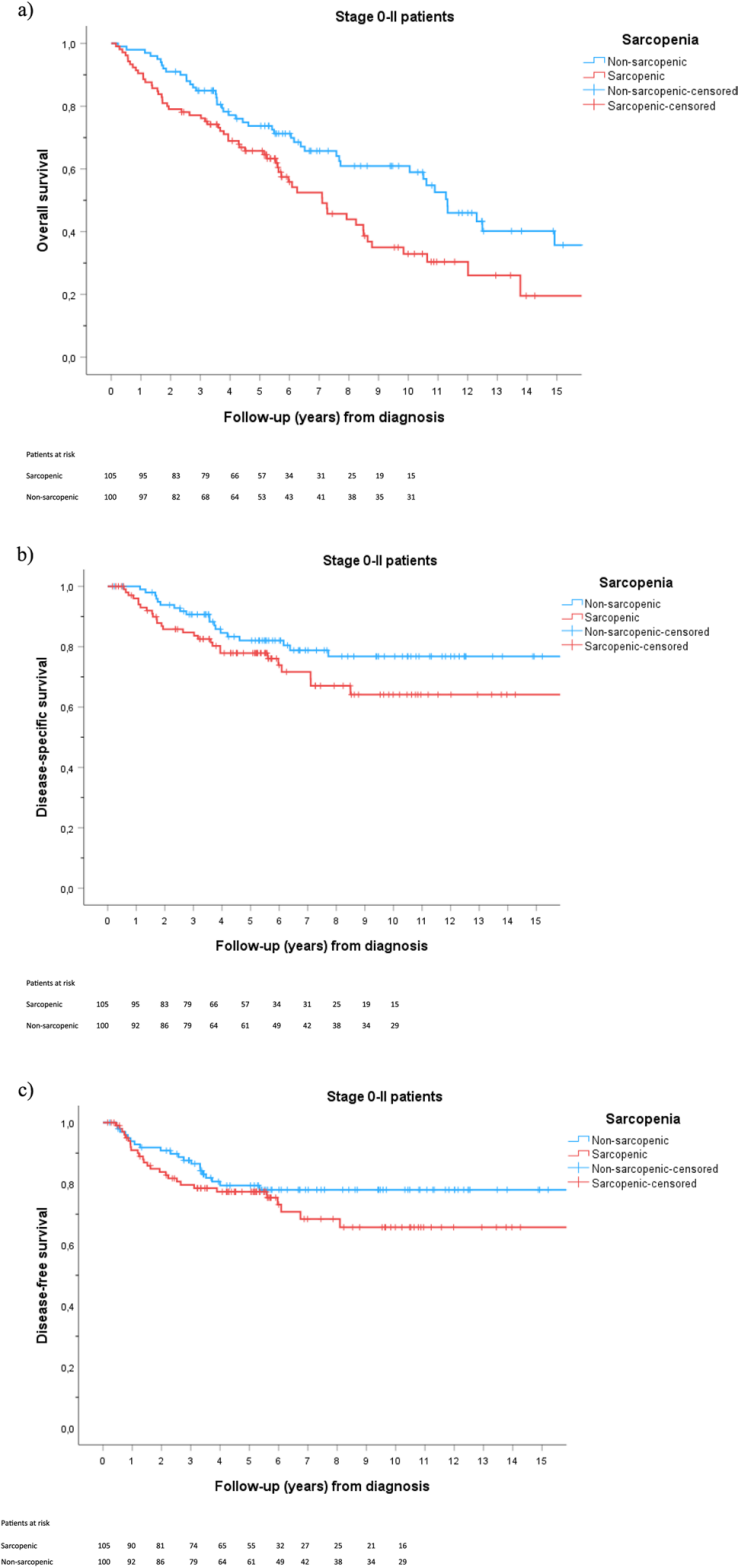
Kaplan-Meier survival of stage 0-II patients (**a**) Overall survival (OS, *P* = 0.007); (**b**) disease-specific survival (DSS, *P* = 0.113) and (**c**) disease-free survival (DFS, *P* = 0.206). Number-at-risk tables are displayed below the X-axis

Kaplan–Meier estimates for stage 0–II patients are shown in Fig. 2. The median OS was 7.1 years (95% CI 5.3–8.9) in sarcopenic patients and 11.3 years (95% CI 9.7–13.0) in non-sarcopenic patients (*P* = 0.007). Five- and ten-year OS were 65.7% and 32.9% in sarcopenic patients compared with 73.7% and 60.9% in non-sarcopenic patients, respectively. No significant differences were seen in DSS or DFS.

## Discussion

The study groups were well balanced in terms of baseline characteristics, operative details, postoperative outcomes, and pathological data. No significant differences were observed in morbidity or short-term mortality between sarcopenic and non-sarcopenic patients. However, long-term survival analysis demonstrated poorer outcomes particularly in stage 0-II sarcopenic patients, with significantly reduced overall survival.

Sarcopenic patients were slightly older than their non-sarcopenic counterparts; however, this difference was not statistically significant. Additionally, sarcopenia was more prevalent among male patients, this finding is consistent with previous meta-analysis [20]. The overrepresentation of male patients in the sarcopenic group is likely multifactorial, potentially driven by a more pronounced age-related decline in muscle mass compared to females. Men generally experience a steeper reduction in testosterone levels with ageing, a hormone that plays a critical role in muscle protein synthesis and maintenance [21]. This hormonal decline may contribute to accelerated muscle loss in male patients.

Additionally, physical activity levels tend to decline more sharply in men after retirement, whereas women often maintain higher levels of daily functional activity, which may help preserve muscle mass. Lifestyle factors, including higher rates of smoking, alcohol consumption, and poorer dietary habits, which are more prevalent among men, may further contribute to this disparity. Furthermore, comorbidities known to promote muscle loss, such as cardiovascular disease, chronic kidney disease, and diabetes, are more frequently observed in males, increasing the risk of sarcopenia [22]. Taken together, these factors likely explain the higher proportion of sarcopenic patients in the male population of this study.

A recent meta-analysis of 42 studies including 11,981 patients found increased short-term mortality, higher complication rates, and poorer OS and DFS in sarcopenic patients [23]. In contrast, our cohort did not demonstrate higher perioperative risk, which may reflect strict perioperative optimisation, patient selection, and uniform surgical protocols in this tertiary setting. While sarcopenia is linked to worse long-term survival. Selection bias likely contributed in this real-world setting, as only patients fit for surgery were included, while patients with severe sarcopenia or frailty, who were at higher risk, were excluded, potentially underestimating its actual impact. Our study examined stage-specific effects, indicating that sarcopenia adversely affected survival in stage 0–II disease, whereas in advanced stages prognosis was dominated by tumour burden.

Tumour location differed between the groups, with sarcopenic patients more often presenting with tumours in the upper stomach. This anatomical distribution likely contributed to the higher proportion of total gastrectomies in sarcopenic patients, although the difference did not reach statistical significance. Total gastrectomy is typically required for tumours in the proximal stomach or those involving the entire gastric body, and this more extensive resection may adversely affect long-term nutritional status. In sarcopenic patients, who already have compromised muscle mass, this could partly explain the observed reduction in overall survival.

These patterns remained consistent in the subgroup of patients with stage 0–II disease. Within this subgroup, sarcopenic patients were also more likely to have intestinal-type GC than non-sarcopenic patients. The relationship between sarcopenia, tumour location, and histological subtype may reflect shared risk factors. Proximal tumours can impair oral intake, contributing to malnutrition and muscle loss. Intestinal-type gastric cancer tends to develop slowly and is more prevalent in older patients, who are also more likely to be sarcopenic. Furthermore, the prolonged disease course of intestinal-type cancer may itself promote progressive muscle wasting.

Patients in both groups received neoadjuvant treatment at comparable rates, consisting exclusively of EOX chemotherapy. This suggests that fitter sarcopenic patients were able to undergo standard treatment protocols at our institution. Previous studies have reported that sarcopenic patients may have reduced tolerance to adjuvant therapy, potentially impacting long-term survival [5, 7]. However, in our cohort, we observed no significant difference in the proportion of patients receiving adjuvant treatment postoperatively.

Adjuvant treatment improved survival significantly in our study. Patients who received preoperative EOX typically continued with the same regimen postoperatively. In cases of poor treatment tolerance, less toxic alternatives were chosen. If disease progression was detected and the patient was able to tolerate further therapy, more effective treatment regimens were selected accordingly. In the early part of the study period, very few patients received adjuvant treatment and were instead admitted to follow-up only; during this time, chemoradiation therapy was also used as a possible adjuvant option.

In the full cohort of 337 patients, sarcopenia at the time of diagnosis was not independently associated with overall, disease-specific, or disease-free survival. Importantly, our interaction analysis showed that the prognostic effect of sarcopenia was not consistent across stages. Sarcopenia was linked to significantly poorer overall survival in patients with stage 0–II disease, whereas no such association was observed in stage III–IV disease.

This stage-dependent effect is biologically plausible. In advanced GC, tumour burden and biology largely dictate survival, which may overshadow the influence of host-related factors such as muscle mass or nutritional status. In contrast, among patients with lower-stage disease—where cancer-specific mortality is less dominant—sarcopenia may indicate reduced physiological reserve, chronic inflammation, and limited recovery capacity, all of which can impair long-term outcomes. Recent studies emphasise the importance of functional measures such as handgrip strength, nutritional optimisation, and multimodal prehabilitation strategies in older surgical patients [24–26], supporting the concept that host factors become particularly relevant once tumour control is achievable. The adverse impact of sarcopenia in lower-stage disease was most evident for overall rather than cancer-specific survival, suggesting that competing risks such as comorbidity and reduced physiological reserve may contribute.

This single-centre study is characterized by its robust long-term follow-up, spanning 20 years and encompassing consecutive selected patients who underwent gastrectomy with curative intent for GC. A key strength of the study is the comprehensive manual review of patient records, rather than reliance on registries, ensuring high-quality data collection. While this approach enhances data accuracy, it also presents a limitation due to its labour-intensive nature.

Nevertheless, the retrospective study design introduces certain inherent limitations, including potential biases such as patient selection and variability in prognostic factors. In subgroup analyses, the number of events was limited, which may have reduced the statistical power of multivariable Cox regression models. Therefore, the data should be interpreted exploratory and further larger prospective studies are needed to confirm these findings. Finally, sarcopenia was defined using CT-based muscle index only, as muscle strength measures and nutritional status were not available.

## Conclusions

Sarcopenia was common among patients undergoing gastrectomy for GC, particularly male patients. Although it was not an independent prognostic factor in the overall cohort, interaction analysis revealed that sarcopenia was associated with significantly poorer long-term survival in patients with stage 0–II disease, whereas no such association was seen in stage III–IV disease. Despite similar rates of perioperative treatment, operative outcomes, and postoperative complications, sarcopenic patients experienced inferior overall survival in lower-stage disease. These findings suggest that sarcopenia is a clinically relevant prognostic factor, even in patients otherwise considered to have a favourable prognosis. Routine assessment of sarcopenia may help identify high-risk patients who could benefit from early nutritional and physical rehabilitation interventions to improve long-term outcomes. Future prospective trials are needed to determine whether such targeted interventions can modify survival in sarcopenic patients, particularly those with early-stage disease.

## Supplementary Information

Supplementary Material 1.

Supplementary Material 2.

Supplementary Material 3.

## Data Availability

The datasets used and analysed in this study are securely stored on a protected server at Helsinki University Hospital. Due to institutional privacy regulations, these data are not publicly accessible. However, they may be made available upon reasonable request to the corresponding author, subject to obtaining the necessary permissions.

## Abbreviations

GC: Gastric Cancer
OS: Overall Survival
CCI: Comprehensive Complication Index
DSS: Disease-specific Survival
DFS: Disease-free Survival
HUH: Helsinki University Hospital
ICD-10: International Classification of Diseases, 10th Revision
ASA: American Society of Anesthesiologists
ICU: Intensive Care Unit
AJCC TNM: American Joint Committee on Cancer classification of malignant tumours
CEA: Carcinoembryonic Antigen
CA19-9: Cancer Antigen 19 − 9
ROI: Region Of Interest
HU: Hounsfield Unit
TMA: Total Muscle Area
SMI: Skeletal Muscle Index
EOX: Epirubicin, Oxaliplatin and Capecitabine
cN+: Lymph Node Positive
FLOT: Fluorouracil, Leucovorin, Oxaliplatin and Docetaxel
SD: Standard Deviations IQR: Interquartile Range
HR: Hazard Ratio
CI: Confidence Interval
BMI: Body Mass Index
Hb: Haemoglobin
Alb: Albumin
