# Supplementary material for: Sarcopenia predicts poor long-term survival but not postoperative complications in gastric cancer surgery: an 18-year retrospective cohort study
Source: World J Surg Oncol. 2025 Dec 2;24:22. doi: 10.1186/s12957-025-04120-6 (PMC12777426; doi:10.1186/s12957-025-04120-6)
Supplement: Supplementary file 1 — Supplementary Material 1. [file 12957_2025_4120_MOESM1_ESM.docx]

| Supplementary table 1. Subanalysis of stage 0, I and II patients | | | |
| --- | --- | --- | --- |
|  | Sarcopenic N=105 | Non-Sarcopenic N=100 | *P* |
| Median age, in years (IQR) | 70.1 (62.6-77.1) | 66.5 (57.6-75.9) | 0.064 |
| Sex, male (%) | 69 (65.7) | 39 (39.0) | <0.001 |
| ASA physical status, n (%) |  |  | 0.376 |
| 1 | 4 (3.8) | 8 (8.0) |  |
| 2 | 26 (24.8) | 31 (31.0) |  |
| 3 | 62 (59.0) | 50 (50.0) |  |
| 4 | 13 (12.4) | 11 (11.0) |  |
| Charlson comorbidity index, n (%) |  |  |  |
| mild (0–2) | 53 (50.5) | 57 (57.0) | 0.401 |
| moderate (3–4) | 39 (37.1) | 35 (35.0) | 0.773 |
| Severe (≥5) | 13 (12.4) | 8 (8.0) | 0.361 |
| Mean Charlson comorbidity index (±SD) | 3.0 (1.4) | 2.7 (1.1) | 0.212 |
| Neoadjuvant treatment (%) | 35 (33.3) | 28 (28.0) | 0.408 |
| Type of resection, n (%) |  |  | 0.258 |
| Total gastrectomy | 49 (46.7) | 38 (38.0) |  |
| Distal gastrectomy | 56 (53.3) | 62 (62.0) |  |
| Approach, n (%) |  |  | 0.812 |
| Open | 96 (91.4) | 90 (90.0) |  |
| Laparoscopic | 9 (8.6) | 10 (10.0) |  |
| Lymph node dissection, n (%) |  |  | 0.504 |
| D0 | 0 | 1 (1.0) |  |
| D1 | 15 (14.3) | 13 (13.0) |  |
| D1+ | 12 (11.4) | 7 (7.0) |  |
| D2 | 78 (74.3) | 79 (79.0) |  |
|  |  |  |  |
| Time median, in minutes (IQR) | 201.0 (166.5-239.0) | 204.0 (169.0-240.0) | 0.726 |
| Blood loss (ml), median (IQR) | 500.0 (262.5-900.0) | 450.0 (200.0-700.0) | 0.220 |
| Splenectomy | 25 (23.8) | 21 (21.0) | 0.738 |
| Reconstruction, n (%) |  |  | 0.091 |
| Roux-en-Y | 54 (51.4) | 46 (46.0) |  |
| Billroth I | 6 (5.7) | 1 (1.0) |  |
| Billroth II | 45 (42.9) | 53 (53.0) |  |
|  |  |  |  |
| Clavien–Dindo grade, n (%) ** |  |  | 0.874 |
| 0 | 12 (11.4) | 18 (18.0) |  |
| 1 | 28 (26.7) | 27 (27.0) |  |
| 2 | 43 (41.0) | 35 (35.0) |  |
| 3a | 13 (12.4) | 11 (11.0) |  |
| 3b | 3 (2.9) | 3 (3.0) |  |
| 4a | 4 (3.8) | 4 (4.0) |  |
| 4b | 0 | 1 (1.0) |  |
| 5 | 2 (1.9) | 1 (1.0) |  |
| CCI, median (IQR) | 22.6 (8.7-31.4) | 22.6 (8.7-32.0) | 0.501 |
| Reoperation, n (%) | 6 (5.7) | 5 (5.0) | 1.000 |
| ICU admission, n (%) | 12 (11.4) | 11 (11.0) |  |
| Length of hospital stay (days), median (IQR) | 8.0 (7.0-11.0) | 8.0 (7.0-11.0) | 0.710 |
| Readmission, n (%) | 6 (5.7) | 10 (10.0) | 0.303 |
| 30-day mortality, n (%) | 2 (1.9) | 1 (1.0) | 1.000 |
| 90-day mortality, n (%) | 3 (2.9) | 1 (1.0) | 0.622 |
|  |  |  |  |
| Histology, n (%) |  |  | 0.032 |
| Intestinal | 47 (44.8) | 28 (28.0) |  |
| Diffuse | 46 (43.8) | 61 (61.0) |  |
| Mixed / other | 12 (11.4) | 11 (11.0) |  |
| Tumour location, n (%) |  |  | 0.005 |
| Lower (antrum, angulus) | 38 (36.2) | 45 (45.0) |  |
| Middle (body) | 51 (48.6) | 53 (53.0) |  |
| Upper (fundus, cardia) | 10 (9.5) | 2 (2.0) |  |
| Other | 6 (5.7) | 0 |  |
| Lymph node status, n (%) |  |  |  |
| Positive | 31 (29.5) | 31 (31.0) | 0.880 |
| R class, n (%) |  |  | 0.498 |
| R0 | 103 (98.1) | 100 (100.0) |  |
| R1 | 2 (1.9) | 0 |  |
| Adjuvant treatment | 33 (31.4) | 26 (26.0) | 0.398 |
