# Supplementary figures and images for: Sarcopenia predicts poor long-term survival but not postoperative complications in gastric cancer surgery: an 18-year retrospective cohort study
Source: World J Surg Oncol. 2025 Dec 2;24:22. doi: 10.1186/s12957-025-04120-6 (PMC12777426; doi:10.1186/s12957-025-04120-6)

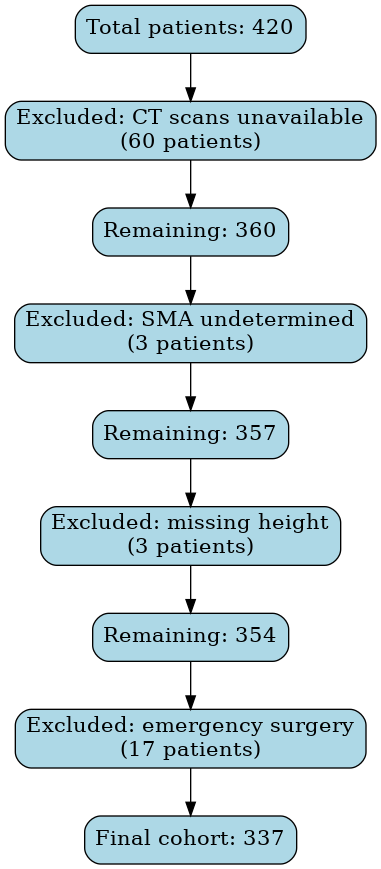

Supplement: Supplementary file 2 — Supplementary Material 2. [file 12957_2025_4120_MOESM2_ESM.docx]
