# Supplementary material for: Sarcopenia predicts poor long-term survival but not postoperative complications in gastric cancer surgery: an 18-year retrospective cohort study
Source: World J Surg Oncol. 2025 Dec 2;24:22. doi: 10.1186/s12957-025-04120-6 (PMC12777426; doi:10.1186/s12957-025-04120-6)
Supplement: Supplementary file 3 — Supplementary Material 3. [file 12957_2025_4120_MOESM3_ESM.docx]

Supplementary Material 2.

a)


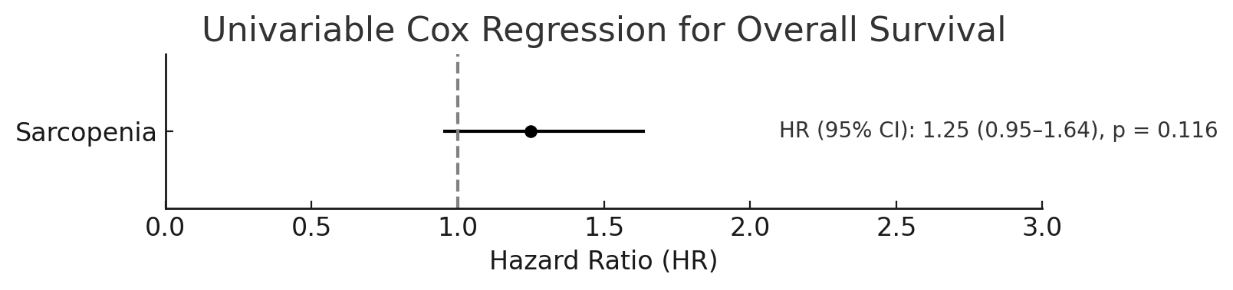


b)


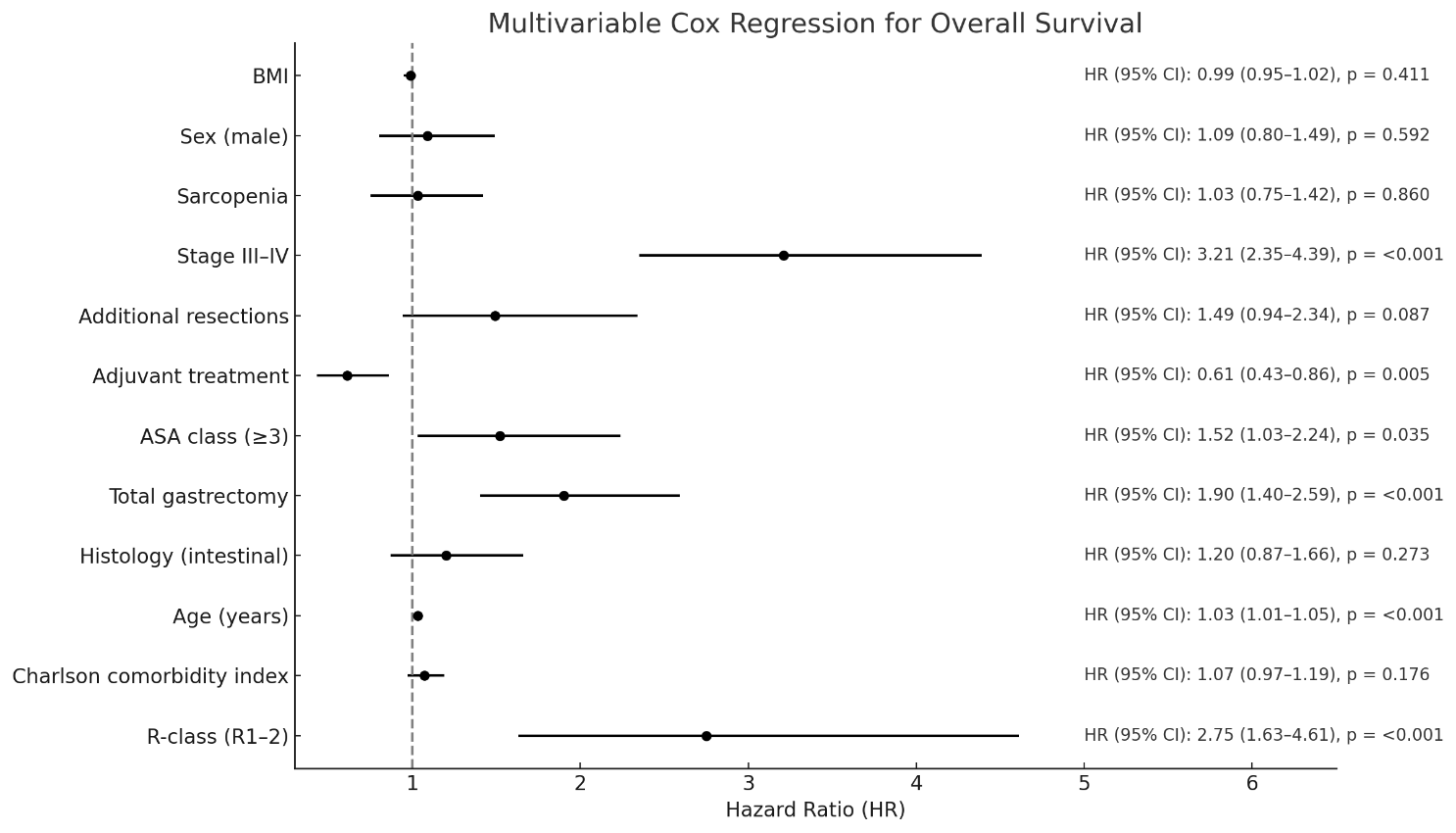


c)


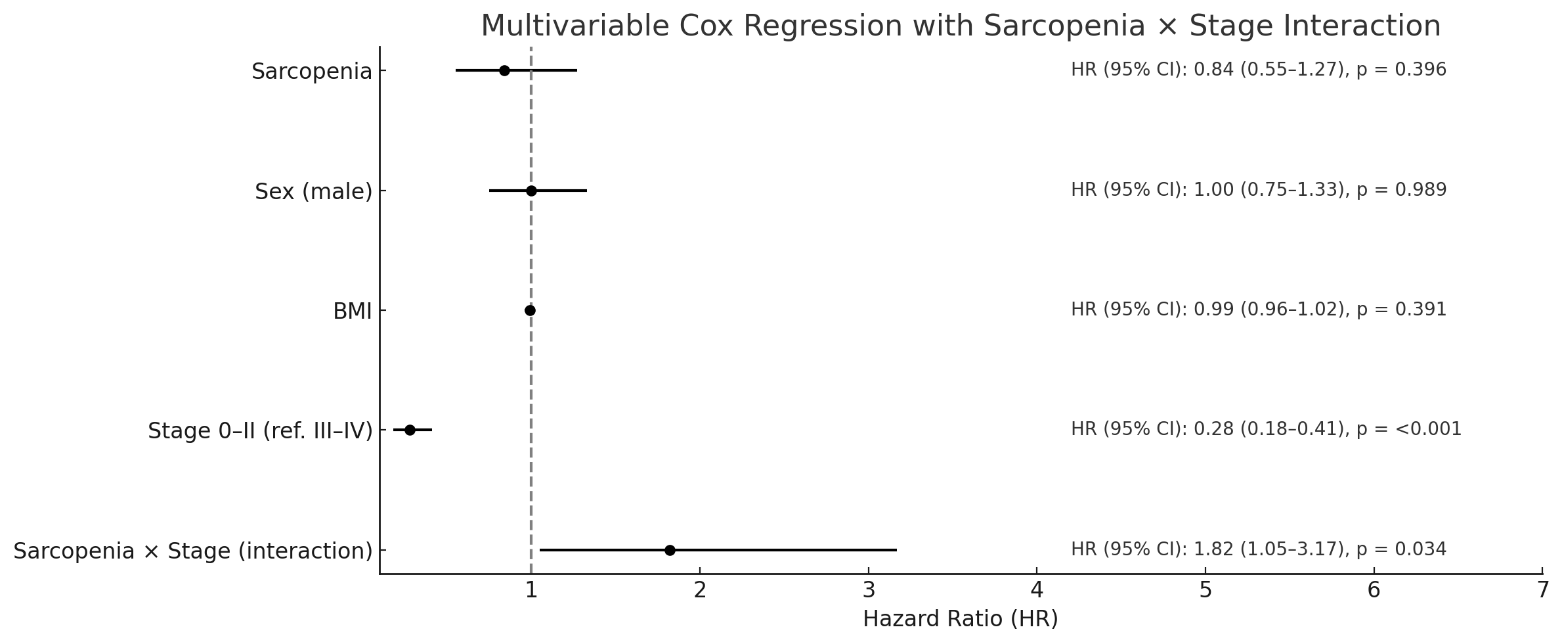


Supplementary Material 2. Forest plot of Cox regression analyses for overall survival: a) univariable; b) multivariable; and c) multivariable Cox regression analysis with stage interaction analysis for overall survival. The plot shows hazard ratios (HR) with 95% confidence intervals (CI) for each variable. The dashed vertical line at HR = 1 indicates no association with survival.
